# miRNA-29 aggravates myocardial infarction via inhibiting the PI3K/mTOR/HIF1α/VEGF pathway

**DOI:** 10.18632/aging.203997

**Published:** 2022-04-04

**Authors:** Xiaoxi Wang, Yanning Liu, Huiqing Hou, Weihua Shao, Dai Huang, Zhihua Hao, Hongyuan Xue, Yuquan Ye

**Affiliations:** 1 Department of Medical Examination Center, Hebei Medical University, Hebei General Hospital, Shijiazhuang 050051, Hebei, China; 2 Department of Neurosurgery, 980 Hospital of PLA Joint Logistics Support Forces, Shijiazhuang 050051, Hebei, China; 3 Medical Examination Center, Hebei General Hospital, Shijiazhuang 050051, Hebei, China; 4 Department of Second Division of Geriatrics, Hebei General Hospital, Shijiazhuang 050051, Hebei, China; 5 Department of Medical Imaging and Ultrasound, Hebei Medical University, Hebei General Hospital, Shijiazhuang 050051, Hebei, China; 6 Ultrasound Department, Hebei Medical University, Hebei General Hospital, Shijiazhuang 050051, Hebei, China

**Keywords:** myocardial infarction, bioinformatics, miRNA-29, PI3K/mTOR/HIF1α/VEGF pathway

## Abstract

Introduction: MI is defined by the presence of myocardial necrosis, which is caused by acute and persistent ischemia and hypoxia of the coronary artery. In recent years, its incidence rate has been on the rise in China.

Methods: GSE34198, GSE97320 and GSE141512 datasets were download for DEG analysis. KEGG pathway analysis, GO analysis, GSEA and PPI network construction were performed. Later, target genes of candidate miRNAs were predicted. Next, echocardiography was conducted to detect the effects of miR-29 on left ventricular structure and cardiac function *in vivo*, and H&E staining was adopted to study the effects of miR-29 on angiogenesis and fibrosis *in vivo*. Furthermore, Western blotting was employed to investigate the effects of miR-29 inhibition on the expressions of proteins related to the PI3K\mTOR\ HIF-1α\VEGF pathway.

Results: There were 162 DEGs involved in MI. GO analysis revealed that inflammatory responses, negative regulation of apoptosis and innate immune response were the main enriched biological processes. KEGG analysis manifested that DEGs were mainly enriched in the PI3K/Akt signaling pathway, and GSEA demonstrated that they were mainly enriched in the PI3K/Akt/mTOR, HIF and VEGF pathways. Moreover, target gene prediction showed that miR-29 was lowly expressed in MI. According to Masson's trichrome staining, miR-29 inhibition promoted angiogenesis, reduced fibrosis, and increased the protein expressions of p-PI3K, p-mTOR, HIF-1α, and VEGF.

Conclusions: MiR-29 may play an important role in the growth and development of MI. After inhibition of miR-29, the PI3K/mTOR/HIF-1α/VEGF pathway is activated to alleviate MI.

## INTRODUCTION

Myocardial infarction (MI) is defined by the presence of myocardial necrosis caused by acute and persistent ischemia and hypoxia of the coronary artery. Clinically, the disease is commonly manifested as acute and persistent retrosternal pain, which cannot be effectively alleviated by rest and nitric acid ester drugs. With the increase of the myocardial enzyme level in serum and the change of progressive electrocardiogram, MI may be complicated with often life-threatening arrhythmia, shock or heart failure [1]. The disease is the most common in Europe and America. About 1.5 million heart attacks occur in the United States each year [2]. Recent years have witnessed a sharp increase in cases of MI in China, with at least 500,000 new births. Its annual incidence rate in males and females is 71‰ *vs.* 22‰ in the 35-84-year-old population, 91‰ *vs.* 25‰ in the 55-64-year old population, 119‰ *vs.* 51‰ in 65-74-year-old population, and 168‰ *vs.* 90‰ in the 75-84-year-old population [3, 4]. However, the pathogenesis of MI remains not clear. MI in most patients develops from coronary atherosclerotic stenosis. Induced by some factors, coronary atherosclerotic plaques rupture, and blood platelets gather on the surface of the ruptured plaque, forming blood clots (thrombi). Then these blots suddenly block the coronary lumen, leading to myocardial ischemia and necrosis. Additionally, a sharp increase in myocardial oxygen consumption or coronary artery spasm also can induce acute MI (AMI). It can be concluded that the disease may be related to genetic factors, chromosomal abnormalities, gene fusion and other elements [5, 6]. Therefore, it is of great significance to explore the molecular mechanism of MI.

Medical bioinformatics is an interdisciplinary discipline that uses computer science as a tool to store, retrieve, analyze and interpret biological and medical data. In recent years, with the rapid development of microarray and high-throughput sequencing technology, the whole transcriptome and genome can be quickly analyzed, which profoundly promotes the management and marketing of life sciences. As research on the gene expression profile of MI has progressed and various public databases have been established, further research on MI can be conducted through bioinformatics, contributing to further investigation of the pathological mechanism of MI [7].

Micro ribonucleic acid (MiR)-29 is associated with cell proliferation, differentiation, metabolism, senescence and apoptosis [8], and it may be a molecular target for disease therapy [9]. Previous studies have confirmed that miR-29 is involved in diseases of the circulatory system, and it can regulate the remodeling process of cardiomyocytes and inhibit cell damage. The phosphoinositide 3 kinase (PI3K)\mammalian target of rapamycin (mTOR)\hypoxia-inducible factor-1α (HIF)-1α\vascular endothelial growth factor (VEGF) signaling pathway is widely involved in many biological processes such as cell proliferation, differentiation, apoptosis, autophagy and metabolism. A large number of studies have shown that various natural active ingredients exert antitumor effects by targeting the PI3K/mTOR/HIF-1α/VEGF signaling pathway, and targeted autophagy induced by this pathway can be used as an important means of treating tumors and increasing the sensitivity of chemotherapy [10]. However, the relationship between this pathway and MI is unclear.

In this study, therefore, bioinformatics technology was applied to excavate the core genes in MI tissues and normal tissues, and enrichment and pathway analyses were performed. In addition, the significant roles of miR-29 and the PI3K/mTOR/HIF-1α/VEGF pathway in MI were verified using public datasets, which were then verified by basic cell experiments.

## RESULTS

### Screening results of DEGs

**Figure 1:**
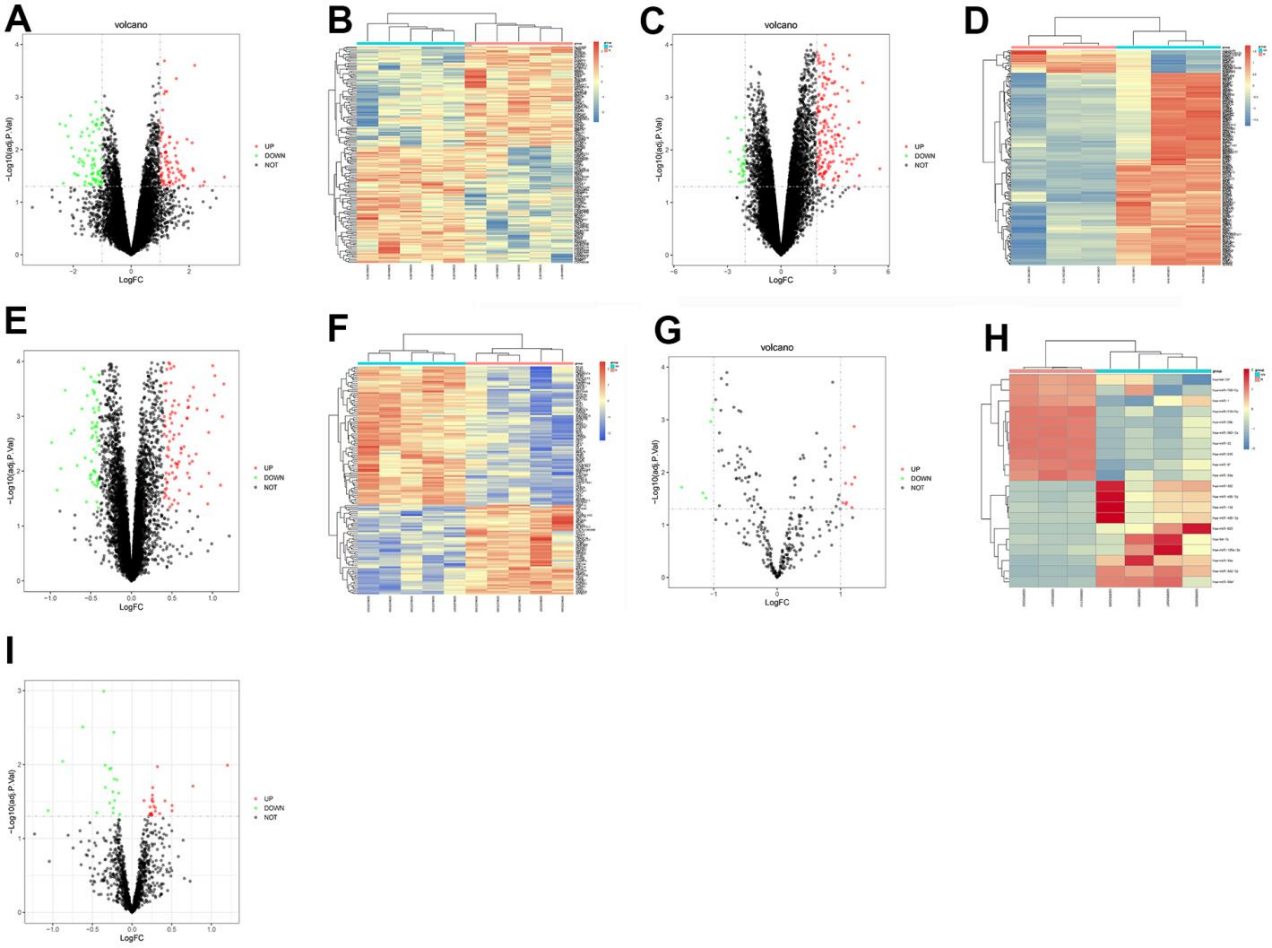
**The screening of DEGs involved in MI.** The volcano maps and heatmaps manifested the DEGs from GSE34198 (**A**, **B**), GSE97320 (**C**, **D**), GSE141512 (**E**, **F**), GSE24548 (**G**, **H**) and GSE76604 (**I**).

Three MI-related datasets, GSE34198, GSE97320and GSE141512, and two miRNA datasets, GSE24548 and GSE76604, were downloaded from GEO database, and quantile normalization was performed. With P<0.05 and |logFC|>1 as the threshold criteria, the screening results showed that there were 162 DEGs in the mRNAs involved in MI in GSE34198, among which 87 were up-regulated and 75 were down-regulated. The ggplot2 package of R software was used to plot the visually clustered volcano maps of DEGs from GSE24548 (Figure 1A), and the pheatmap package of R software was used to draw the clustered heatmaps of DEGs (Figure 1B). In the same way, the visually clustered volcano maps of DEGs and clustered heatmaps of DEGs in GSE97320 (Figure 1C, 1D), in GSE141512 (Figure 1E, 1F), in GSE24548 (Figure 1G, 1H) and in GSE141512 (Figure 1I) were drawn.

### Bioinformatics analysis results

**Figure 2:**
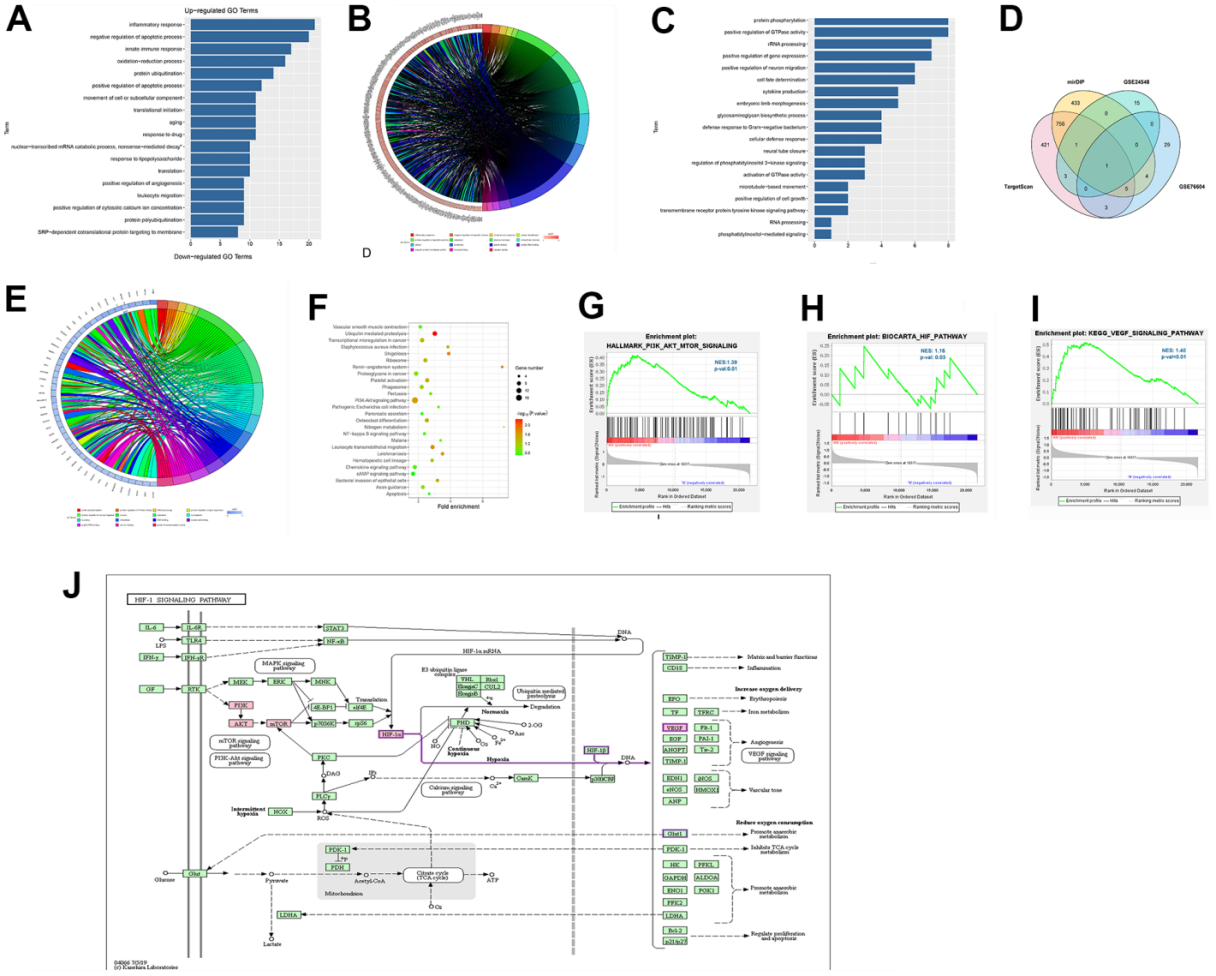
**Enrichment analysis by GO and KEGG.** (**A**, **B**) The enriched terms of up-regulated pathways. (**C**, **D**) The enriched terms of down-regulated pathways. (**E**) The KEGG pathway diagram showed the PI3K/Akt signaling pathway and other pathways. (**F**–**I**) GSEA showed the enrichment of DEGs in the PI3K/Akt/mTOR pathway, HIF pathway and VEGF pathway. (**J**) KEGG online tool query showed that PI3K/AKT/mTOR was correlated with HIF and VEGF.

The common DEGs were screened by RRA package (|logFC|<1, P<0.05), and the enrichment of DEGs was assessed by GO analysis and KEGG analysis. Using DAVID, the enrichment of the corresponding DEGs in biological processes was analyzed to integrate GO terms and create a biological process network of DEGs. The up-regulated pathways (Figure 2A, 2B) and down-regulated pathways (Figure 2C, 2D) of the DEGs were plotted by R software. From the GO pathway diagram, inflammatory responses, negative regulation of apoptosis, innate immune responses and other up-regulated pathways and protein phosphorylation, positive regulation of GTPase activity, rRNA processing and other down-regulated pathways were found to be the enrichment pathways of DEGs involved in MI. KEGG pathway analysis of DEGs was conducted, and the KEGG pathway diagram was plotted (Figure 2E), which showed that DEGs were enriched in the PI3K/Akt signaling pathway and other pathways. In addition, GSEA showed the enrichment of DEGs involved in the PI3K/Akt/mTOR pathway, HIF pathway and VEGF pathway (Figure 2F–2I). KEGG online tool query showed that the PI3K/AKT/mTOR pathway was correlated with HIF and VEGF pathways (Figure 2J).

### PPI network analysis results

**Figure 3:**
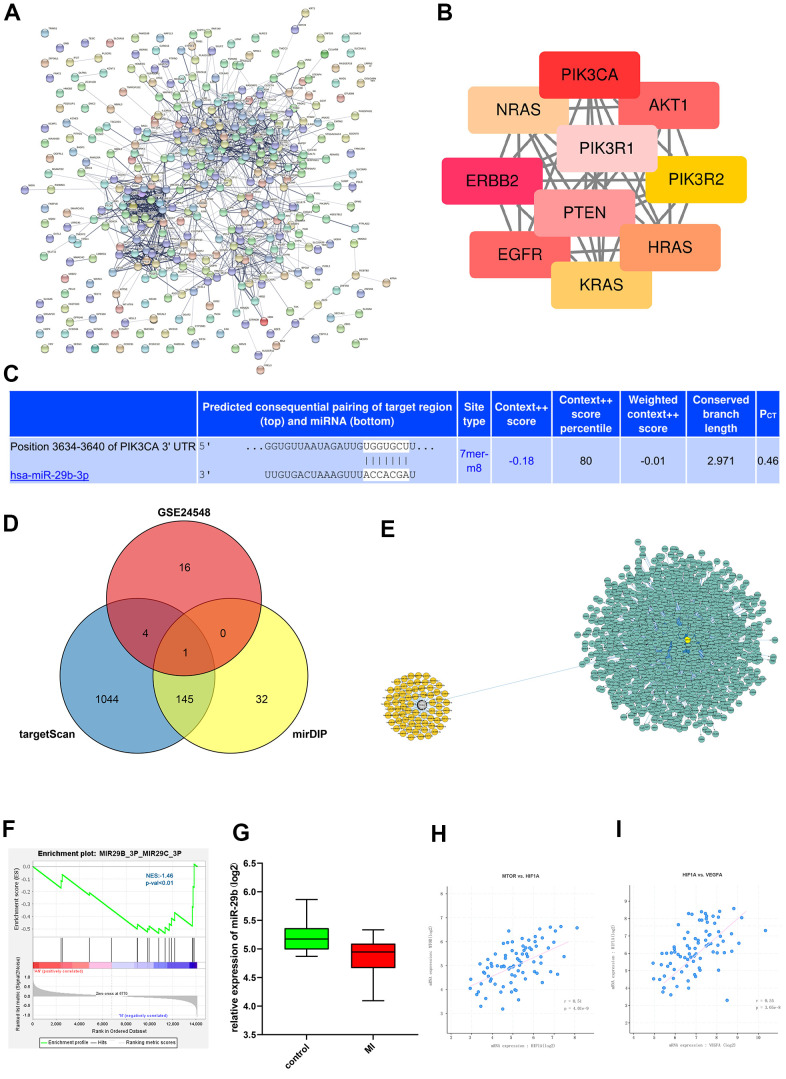
**PPI network analysis and miRNA target gene prediction.** (**A**) PPI network of the DEGs. (**B**) The hub genes identified from the PPI network. (**C**) The binding sites of mRNAs and miRNAs were plotted. (**D**) Venn diagram for intersection of DEGs from GSE24548 and GSE76604 showed the miRNAs such as miR-29b. (**E**) miRNA and mRNA interaction network. (**F**) Through GSEA, we found that miR-29 was enriched. (**G**) The expression of miR-29 was lower in MI group. (**H**, **I**) Correlation analyses between mTOR and HIF-1α and between HIF-1α and VEGF indicated that HIF-1α was positively correlated with mTOR and VEGF, respectively.

PPI analysis was then carried out. The enrichment of DEGs involved in GO pathways were analyzed by PPI network. In the PPI network established based on the STRING database using the Cytoscape tool, we found that PIK3CA and other DEGs were the key genes of MI, proving that these genes influence the incidence and progression of MI (Figure 3A, 3B).

### Predicted target genes of miRNAs

According to the gene prediction results, the binding sites of mRNAs and miRNAs were plotted (Figure 3C). The target genes of candidate miRNAs were predicted by TargetScan and mirDIP online tools, and the VennDiagram package was used to draw the Venn diagram for intersection of DEGs from GSE24548 and GSE76604,, which filtered out miRNAs such as miR-29b (Figure 3D). Cytoscape was used to construct the miRNA and mRNA interaction network (Figure 3E). Through GSEA, we found that miR-29 was enriched in normal group (Figure 3F).

### Statistical analysis results of target genes

The content of miR-29 in different groups was analyzed by statistical methods, and it could be seen that the expression of miR-29 was low in MI group and high in control group (Figure 3G). Correlation analyses between mTOR and HIF-1αand between HIF-1α and VEGF indicated that HIF-1α was positively correlated with mTOR and VEGF, respectively (Figure 3H, 3I).

### Effects of miR-29 on left ventricular structure and cardiac function *in vivo*

**Figure 4:**
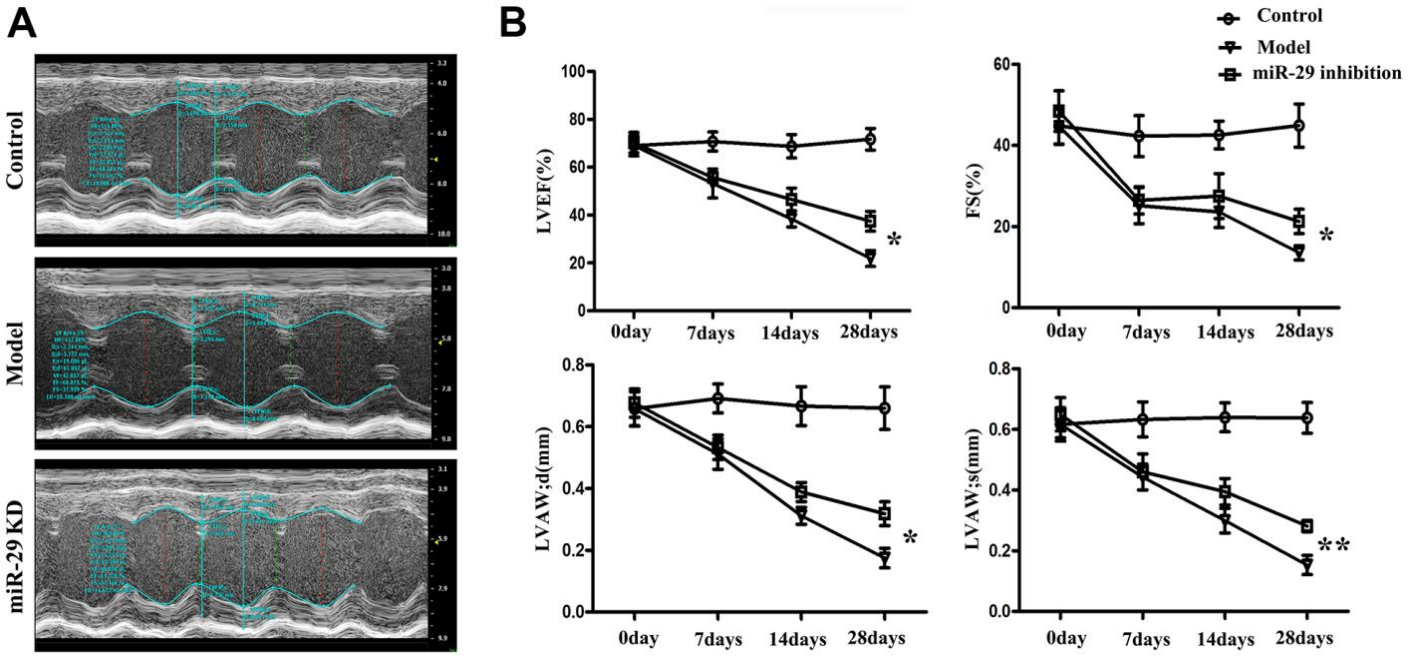
**Effects of miR-29 on cardiac structure and function *in vivo*.** (**A**) Echocardiography results showed that LVEF, FS (%) and LVAW (diastolic and systolic) were gradually decreased in miR-29 inhibition group and model group from 0 d to 28 d, respectively, and these diseases were more significant in model group than those in miR-29 inhibition group. (**B**) Result is displayed in terms of statistical chart. Data were presented as mean ± SE (*P<0.05, **P<0.01). LVEF: left ventricular ejection fraction; LVAW; d: left ventricular anterior wall; diastolic; LVAW; s: left ventricular anterior wall; systolic; FS%: fractional shortening.

Transthoracic echocardiography was performed to assess cardiac structure and function. Echocardiography showed changes in left ventricular structure and cardiac function at the systole and diastole. The red dashed line represents the length of left ventricular internal diameter at diastole; the green dashed line represents the length of left ventricular internal diameter at diastole; the solid blue line shows the length of the left ventricular anterior wall (LVAW) thickness, left ventricular posterior wall (LVPW) thickness (Figure 4A). In miR-29 inhibition group and model group, LVEF, FS (%) and LVAW (diastolic and systolic) were gradually decreased from 0 d to 28 d, respectively (P<0.05; P<0.05; P<0.05; P<0.01; Figure 4B), and the decreases in model group were more obvious than those in miR-29 inhibition group.

### Effects of miR-29 on angiogenesis and fibrosis *in vivo*

**Figure 5:**
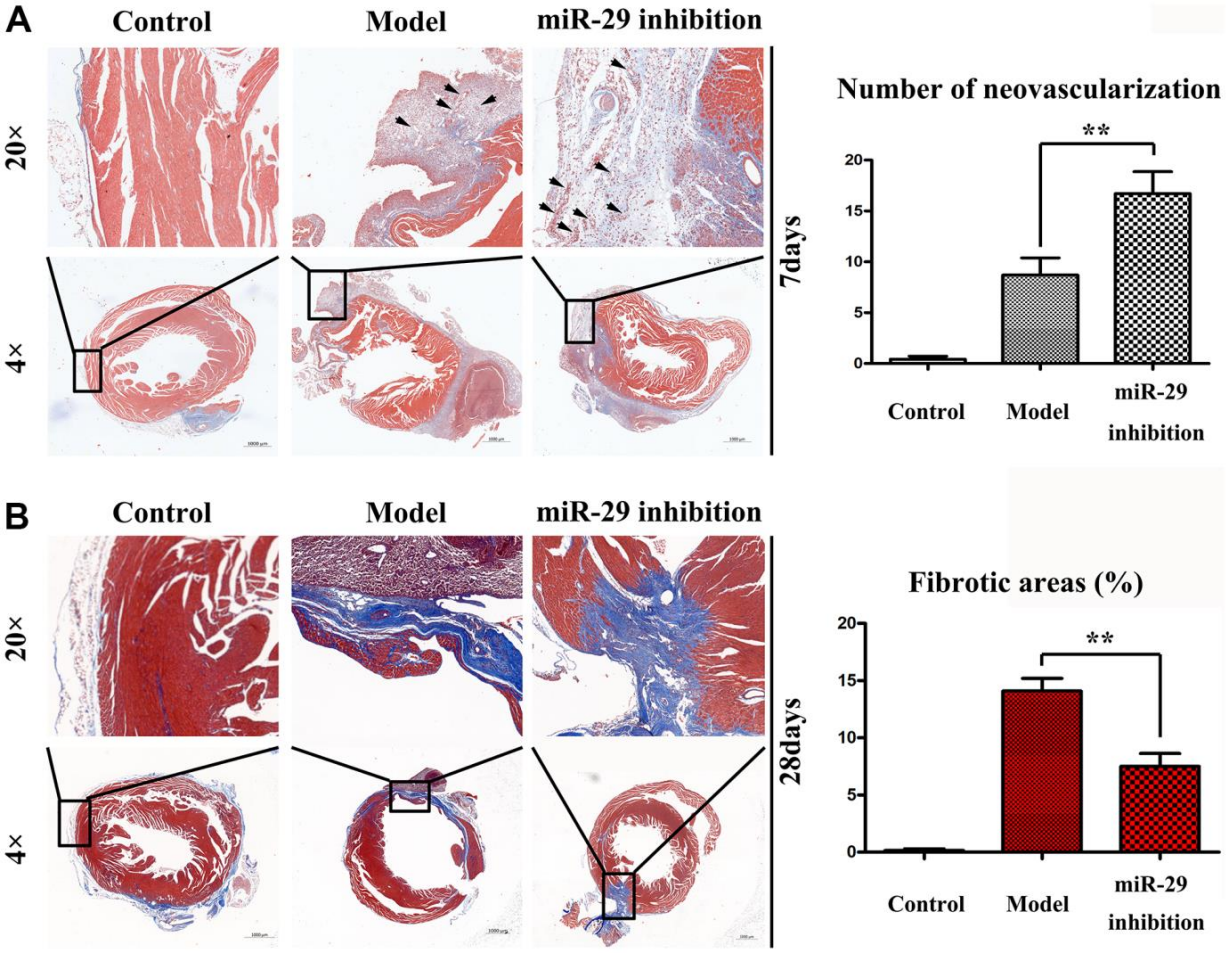
**Effects of miR-29 on angiogenesis and fibrosis *in vivo*.** The vessel sections were stained by hematoxylin and eosin. The section magnification was 4× and 20×, respectively. (**A**) H&E staining showed a higher degree of neovascularization (arrows) in miR-29 inhibition group than that in model group at 7 d, when the section magnification was 4×. (**B**) H&E staining showed less fibrotic areas in miR-29 inhibition group than those in model group at 28 d, when the section magnification was 4×. Results showed that miR-29 OE promoted angiogenesis and reduced fibrosis. Data were presented as mean ± SE (**P<0.01).

The vessel sections were stained by Masson's trichrome staining to identify specific morphologies. The results revealed a higher degree of neovascularization (arrows) in miR-29 inhibition group compared with that in model group at 7 d, when the section magnification was 4× (P<0.01; Figure 5A). Hematoxylin and eosin (H&E) staining results manifested that there were less fibrotic areas in miR-29 inhibition group than those in model group at 28 d, when the section magnification was 4× (P<0.01; Figure 5B). The tissue structure within the boxes were magnified 20×. The above results indicated that miR-29 inhibition promotes angiogenesis and reduces fibrosis.

### Effects of miR-29 inhibition on the expression of proteins involved in the PI3K\mTOR\HIF-1α\VEGF pathway *in vivo*

**Figure 6:**
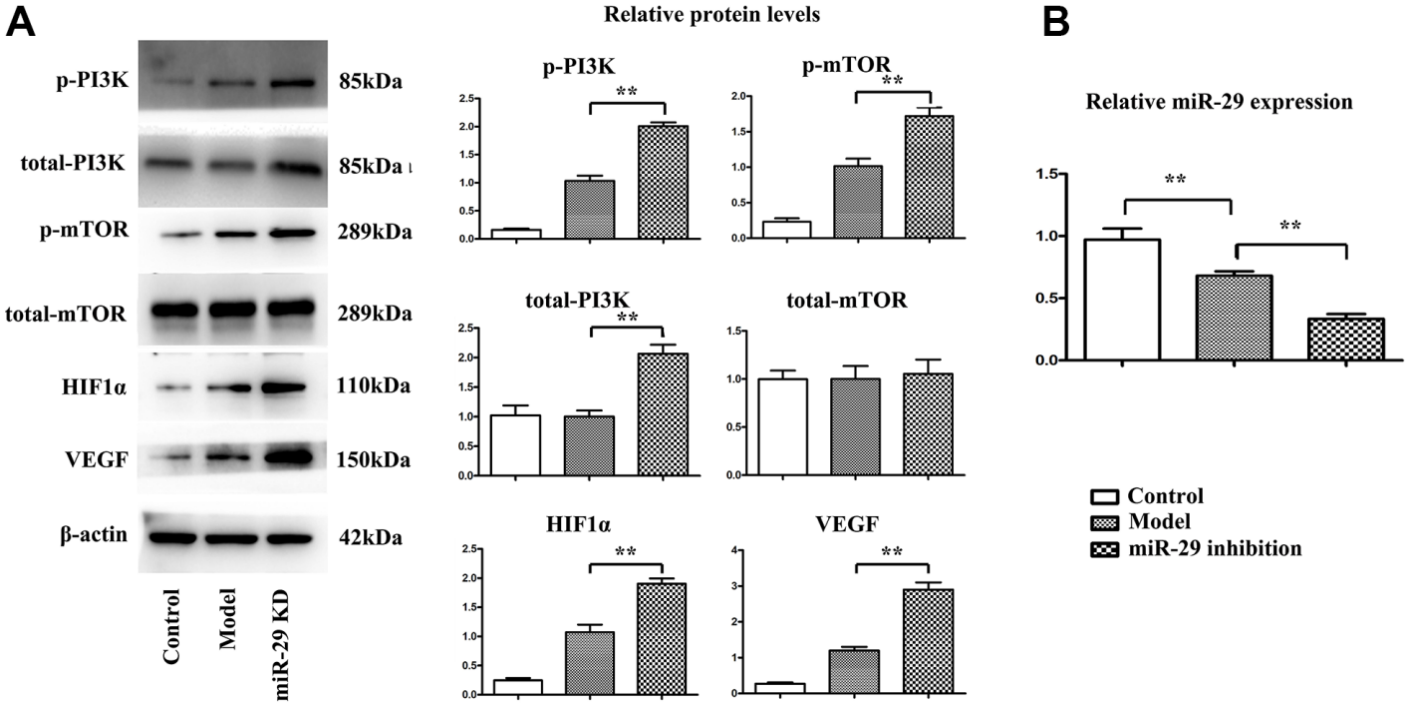
**Effect of miR-29 inhibition on the expressions of proteins involved in the PI3K\mTOR\HIF1α\VEGF pathway *in vivo*.** (**A**) Results showed that the protein expressions of p-PI3K, p-mTOR, HIF-1α, and VEGF were elevated in miR-29 inhibition group compared with those in model group. (**B**) The relative miR-29 fold change was tested by qPCR, and it was found that the miR-29 level was decreased in model group compared with that in control group, and miR-29 inhibition lentivirus group exhibited the lowest miR-29 level among 3 groups. Data were presented as mean ± SE (**P<0.01). PI3K: phosphoinositide 3 kinase; mTOR: mammalian target of rapamycin; HIF-1α: hypoxia-inducible factor-1α; VEGF: vascular endothelial growth factor.

Western blotting assay was conducted to determine whether miR-29 inhibition can affect the expressions of proteins involved in the pathway *in vivo*, with β-actin as a loading control. The integrated ratio of the absorbance area of target protein bands to that of β-actin bands was used to assess the target protein expression level. Results showed that the protein expressions of p-PI3K, p-mTOR, HIF-1α, and VEGF were up-regulated in miR-29 inhibition group compared with those in model group. In addition, the relative miR-29 expression levels were tested by qPCR, which demonstrated that the miR-29 inhibition group showed the lowest expression levels compared with model group and control group, indicating that miR-29 lentivirus effectively suppresses the miR-29 level (P<0.01; Figure 6A, 6B).

### Effects of miR-29 inhibition on the expression of proteins involved in the PI3K\mTOR\HIF-1α\VEGF pathway in endothelial cells

**Figure 7:**
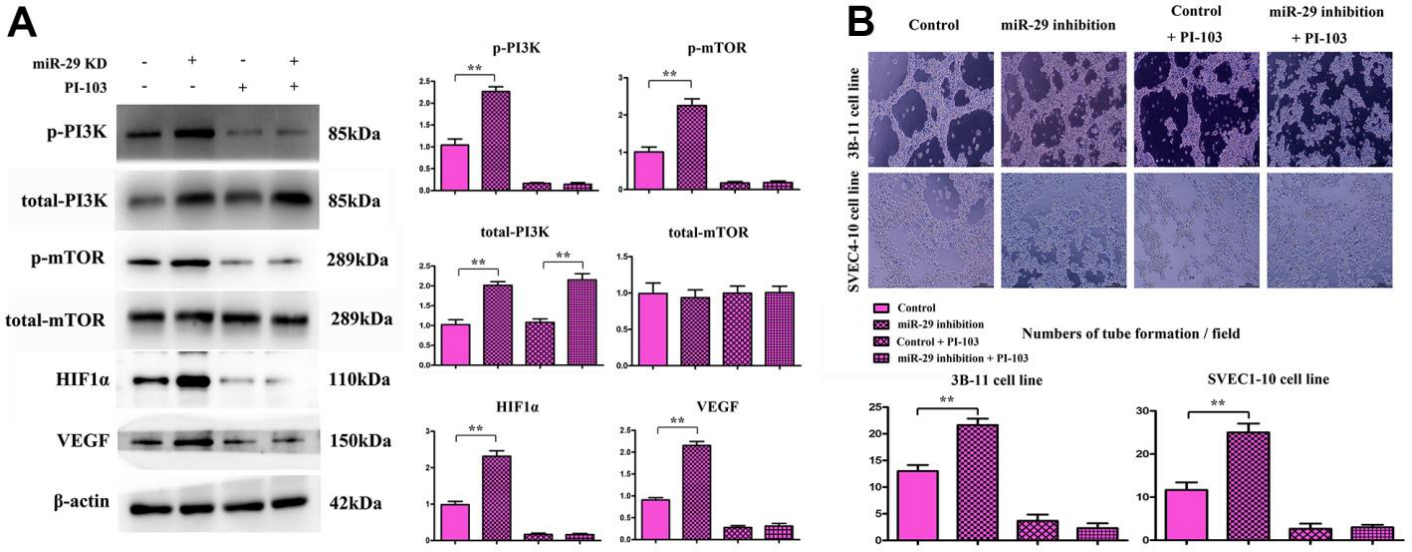
**Effect of miR-29 inhibition on the expressions of proteins involved in the PI3K\mTOR\HIF-1α\VEGF pathway in endothelial cells.** (**A**) Results showed that the protein expressions of PI3K, mTOR, HIF-1α, and VEGF in endothelial cells rose in miR-29 inhibition group compared with those in control group. Besides, (**B**) the endothelial tube formation assay results displayed that miR-29 inhibition increased the number of tubes, and PI-103 (the specific inhibitor for PI3K signals) almost inhibited the tube formation. Data were presented as mean ± SE (**P < 0.01). PI3K: phosphoinositide 3 kinase; mTOR: mammalian target of rapamycin; HIF-1α: hypoxia-inducible factor-1α; VEGF: vascular endothelial growth factor; NC: negative control.

The expression of proteins involved in the PI3K\mTOR\HIF-1α\VEGF pathway in endothelial cells were detected via Western blotting, with β-actin as a loading control. The integrated ratio of the absorbance area of target protein bands to that of β-actin bands was used to assess the target protein expression level. Results showed that the protein expressions of p-PI3K, p-mTOR, HIF-1α, and VEGF *in vitro* rose in miR-29 inhibition group compared with those in control group (P<0.01; Figure 7A). Furthermore, the endothelial tube formation assay results displayed that miR-29 inhibition increased the number of tubes, and PI-103 (the specific inhibitor for PI3K signals) almost inhibited the tube formation (Figure 7B).

## DISCUSSION

MI can bring about the myocardial necrosis in certain areas, triggering acute or chronic heart failure, dyspnea, chest tightness, shortness of breath, inability to lie down in a supine position, but it still can escort for pulmonary heart disease and hypertension. Fatal malignant tachyarrhythmias, such as ventricular tachyarrhythmias, and even ventricular tachycardia in severe cases, can occur after MI due to myocardial necrosis and cardiac conduction dysfunction. After a heart attack, the heart muscle in this area changes, but the systolic blood pressure can still acts on this area and the heart may rupture. Therefore, it is important to study the molecular mechanism of MI. The results of this study mainly revealed that after miR-29 inhibition, the PI3K/mTOR/HIF-1α/VEGF pathway was activated, and the VEGF expression was increased, thus promoting angiogenesis, protecting the myocardium, and alleviating MI.

MiRNAs are a class of conserved small single-stranded non-coding RNAs. MiR-29 is closely associated with heart diseases such as MI, cardiomyocyte hypertrophy and heart failure. A growing number of studies have revealed that miR-29 influences cardiac viability by regulating cardiomyocyte death and regeneration after MI [11–13].

A study has manifested that miRNAs inhibiting angiogenesis mainly include miR-221, miR-222, miR-29b and miR-29c. Therefore, we can promote myocardial angiogenesis by inhibiting miR-29 [6] to treat MI.

It has been recently found that miRNAs regulate the function of cardiomyocytes and play a role in fibrosis and antifibrosis in the pathogenesis of AMI [14]. Other studies have shown that miR-29b can inhibit cardiac fibrosis and improve cardiac function [15]. A multivariate Cox regression analysis was performed to detect the death of patients with AMI during the follow-up period, as well as gender, age, and the expression levels of miR-29b and miR-424 in serum. The results showed that the expression levels of miR-29b and miR-424 in serum were both risk factors for the death of AMI patients (Figures 1, 2).

A study of Wang et al. [16] showed that miR-29b caused cardiovascular endothelial dysfunction through its reaction with PA200 dependent protein kinase. It has been found that miR-29b expression is associated with aldosterone-mediated vascular pathogenesis in vascular smooth muscle cells [17]. It also inhibits cell proliferation and differentiation and induces cell death. In this study, we detected the proliferation in the case of miR-29b overexpression (OE) or inhibition. After the miR-29b level was decreased, aldosterone increased cell proliferation and thus supported the vascular remodeling process. In general, miR-29b appears to have a cell-type-specific and possible stimulus-dependent effect on proliferation, as the oncogenic and tumor-suppressive functions of miR-29b have been proposed [18, 19]. KINET’s study reinforced this theory by analyzing the level of miR-29b in atherosclerosis [20], and the related results were shown in Figures 2, 3.

The increase of the number of coronary artery lesions often represents the increase of the severity of MI in patients. With the increase of the number of coronary artery lesions, the levels of miR-29, H-FABP and CK-MB in patients with MI are significantly increased, suggesting that there is a certain correlation between miR-29 and the severity of MI in patients. This may be because the increased expression of miR-29 *in vivo* can increase the cardiac systolic load, aggravate vascular endothelial injury, cause certain damage to myocardial cells, and hasten the apoptosis of myocardial cells and heart remodeling, thereby increasing the severity of MI in patients [21]. Therefore, it is speculated that miR-29 may play an important role in the growth and development of MI.

The PI3K/mTOR/HIF-1α/VEGF pathway is a classical signaling pathway in angiogenesis. MiR-29c regulates this pathway by negatively regulating target genes SPL, PDPK and IGF-1, and it can not only directly inhibit the expression and activity of eNOS, but also inhibit endothelial cell proliferation, migration and angiogenesis [22, 23]. MiRNAs can directly target VEGFs or regulate VEGF expression through target genes. VEGFs are angiogenic factors, which can increase vascular permeability, and promote extracellular matrix degeneration, vascular endothelial cell migration and proliferation and angiogenesis. In this study, tube formation assay verified this phenomenon and identified the correlations of miR-29 with PI3K-signal activation and angiogenesis in *in-vitro* experiments (Figure 7A, 7B), the miR-29 directly combine to PI3K and resulted the degradation of total-PI3K, thereby, the phosphorylated PI3K level were decreased correspondingly. At the same time, VEGFs can promote angiogenesis and the formation of collateral circulation after infarction, improve the blood and oxygen supply of the myocardium, and increase the survival rate of dying myocardial cells.

A study [24] has shown that ginkgolide B buffers hypoxia-induced miR-29 expression and thus has a cardioprotective effect by inhibiting ischemia-induced apoptosis in MI. Ginkgolide B also triggers the phosphorylation of PI3K/Akt and induces SP1. Later, PI3K/Akt activation increases the activity of cytochrome C oxidase and then attenuates hypoxic apoptosis [25]. However, this beneficial effect influences the survival and apoptosis of cells overexpressing miR-29 to a certain extent. Therefore, ginkgolide B-induced miR-29 inhibition may be the basis of this beneficial effect. In addition, it appeared that both PI3K/Akt and SP1 were inactivated in cardiomyocytes (Figures 4–6). Ginkgolide B prevents signal passivation in response to hypoxic stimulation, and this inhibitory activity depends on miR-29 inhibition. In addition, the targeting effects of miR-29 on PI3K, Akt and SP1 have been identified in fibroblasts [26].

Yumei Ye et al. showed that miR-29a and miR-29c may potentially modulate ischemia-reperfusion injury [27]. Potential targets for miR-29 included Mcl-1 (a member of the antiapoptotic Bcl-2 family) [28], p85a (a regulatory subunit of PI3K), and cell division cycle 42 (CDC42) [27]. When miR-29 expression was down-regulated by miR-29 inhibitors or pioglitazone, H9c2 cells were protected from ischemia-reperfusion injury, as shown by increased cell survival and decreased caspase-3 activity. The down-regulation of miR-29a or miR-29c by antagonists led to the activation of PI3K, followed by the activation of the pro-survival kinase Akt and the anti-apoptotic mediator Mcl-1, and significantly reduced the size and persistence of MI. The content of miRNAs in circulating blood is closely related to the condition of myocardial damage. When AMI occurs, the patient's myocardium will suffer obvious damage in a short time. Myocardial cells are destroyed and will release many substances into blood, and miRNAs are important indicators for the substances released into blood by the necrotic myocardium.

The above literature review is consistent with the results of this study. After miR-29 inhibition, the PI3K/mTOR/HIF-1α/VEGF pathway was activated, and VEGF expression was increased, thereby promoting angiogenesis, protecting the myocardium, and improving MI, which were verified by *in-vivo* and *in-vitro* experiments (Figures 6, 7).

Despite the rigorous bioinformatics analysis in this paper, there are still some shortcomings. In this study, no human clinical trial was conducted to further clarify the function of miR-29. Therefore, in the future research, we should carry out in-depth exploration in this aspect.

In conclusion, miR-29 may play an important role in the growth and development of MI. After miR-29 inhibition, the PI3K/mTOR/HIF-1α/VEGF pathway is activated, and VEGF expression is increased. VEGF can promote angiogenesis and form collateral circulation after infarction, improve the blood and oxygen supply of the myocardium, and improve the survival rate of dying cardiomyocytes.

## MATERIALS AND METHODS

### Bioinformatics analysis

We searched datasets with the key word "myocardial infarction" from the GENE EXPRESSION OMNIBUS (GEO) database (https://www.ncbi.nlm.nih.gov/gds/), and obtained three datasets, namely, GSE34198, GSE97320 and GSE141512. Besides, we also downloaded two miRNA sequencing datasets, namely, GSE24548 and GSE76604, which contained gene expression profiles of patients with MI. The RNA-seq data were normalized using Limma package of R software, and differentially expressed genes (DEGs) were screened (|logFC|<1, P<0.05). Subsequently, ggplot2 package of R software was used to plot the visually clustered volcano maps of DEGs from GSE34198, GSE97320 and GSE141512, and pheatmap package of R software was utilized to draw the clustered heatmaps of DEGs. Besides, the integrated DEGs were screened using RRA package (|logFC|<1, P<0.05). In the same way, visually clustered volcano maps and heatmaps of DEGs from GSE24548 and GSE76604 were also plotted.

### Functional enrichment analysis

Gene Ontology (GO) enrichment analysis and Kyoto Encyclopedia of Genes and Genomes (KEGG) enrichment analysis were performed for screening of common DEGs from GSE34198, GSE97320 and GSE141512. The Database for Annotation, Visualization and Integrated Discovery (DAVID, https://david.ncifcrf.gov) was used to analyze the relative enrichment of DEGs in biological processes, cell components and molecular functions, to integrate GO terms and create a biological network of DEGs. Next, GOPlot and ggplot2 packages were adopted for GO function and KEGG pathway enrichment analyses of DEGs in R software.

### Protein-protein interaction (PPI) network analysis

The interaction between proteins encoded by DEGs was analyzed using PPI analysis database (https://www.string-db.org/) to construct the PPI network. Later, the PPI network was visualized by Cytoscape, and the strength of correlations among DEGs was determined using the MCODE plug-in.

### GESA

Gene Set Enrichment Analysis (GSEA) tool (http://www.gsea-msigdb.org/) was used for GSEA of all genes, and the corresponding pathway map was drawn.

### Prediction of target genes of miRNAs

The target genes of candidate miRNAs were predicted by TargetScan and mirDIP online tools, and the predicted target genes from GSE24548 and GSE76604 were obtained by Venn diagram, which was drawn using VennDiagram package. After that, the binding sites of mRNAs and miRNAs were plotted according to the gene prediction results.

### Establishment of mouse model of MI

Male C57BL/6J mice aged 6-8 weeks old were purchased from Skbex Biotechnology Co., Ltd. and housed in an environment with a 12-hours light/dark cycle at 22±2° C. The mice in control group received Sham operation but no lentivirus injection, and those in model group and miR-29 inhibition group were intramyocardially injected with miR-29 negative control (NC) and miR-29 inhibition-lentivirus (each mouse was injected with 100 μL of 1×10^8^ PFU/mL lentivirus through the tail vein) at 7 days before MI modeling. The mice were anesthetized with 5% isoflurane, and the skin was cut horizontally along the third to fourth intercostal spaces for heart exposure. After that, left anterior descending (LAD) coronary artery was ligated at 1 mm from the aortic root between the pulmonary conus and the auricula sinistra. The myocardium below the ligation turned pale, and local myocardial movement weakened, suggesting the successful establishment of the MI model. Animal care and experimental procedures were approved by the Institutional Animal Care and Use Committee of Hebei General Hospital.

### Cardiac structure or function detection by echocardiography

Cardiac structure following treatment were evaluated using a high-frequency ultrasound system Vevo 2100 (VisualSonics, Inc., Toronto, ON, Canada) with a 30-MHz central frequency scan head. After the mice were anesthetized, two-dimensional echocardiographic measurements were obtained. The left ventricular end-systolic diameter (LVESd), left ventricular end-diastolic diameter (LVEDd), left ventricular ejection fraction (LVEF) and left ventricular fractional shortening (LVFS) were recorded. Each index was measured for 3 cardiac cycles.

### Masson's trichrome staining

Hearts were collected in different groups, fixed in 4% paraformaldehyde, embedded in paraffin, and cut into 4-μm-thick sections. Subsequently, the sections were conventionally deparaffinized to water, stained with Mordant, and then washed with water. Afterwards, the sections were stained with azure blue staining solution for 2-3 min and then with hematoxylin staining solution for 2-3 min. Later, acid ethanol differentiation solution was used for differentiation for several seconds until the tissues turned red completely. Next, the sections were stained with ponceau fuchsin dye for 5-10 min, followed by differentiation with 1% phosphomolybdic acid aqueous solution for 3-5 min and staining with aniline blue or light green solution for 5 min. Lastly, the sections were blocked with neutral gum and pictured by a microscope.

### Cell culture and miR-29 inhibition lentivirus infection

3B-11 cell line (mouse endothelial cell line) was resuscitated at 37° C, and inoculated in a 25 cm^2^ culture flask with 10% fetal bovine serum + 1% double antibody + 89% DMEM medium. Later, the cells were cultured in a 5% CO_2_ cell incubator at 37° C, and the medium was changed every two days. When the cell fusion reached 90%, the original medium was sucked off, and the cells were washed twice with DPBs, and digested with 1 mL of 0.25% trypsin, Under the microscope, the cells were presented with a spherical shape by shrinking, and then the digestion was terminated by adding 2 mL of complete culture medium. The cells on the wall of the culture flask were gently blown down with a pipette, and the cell suspension was sucked into a 15 mL sterile centrifuge tube. After centrifugation at 1000 rpm for 5 min, the supernatant was removed, and the fresh complete culture medium was added for re-suspension. After repeated blowing into a single cell suspension with a pipette, the cell suspension was subcultured at the ratio of 1: 3. At 18-24 h before infection with miR-29 NC and inhibition lentivirus, 3B-11 cells were placed in 24-well plates at 1×10^5^ cells/well. When the fusion degree of cells reached 50%, 1 mL of fresh medium was used to replace the original medium, in which the cells containing 6 μg/mL Polybrene were cultured. Next, an appropriate amount of virus suspension was added for cell incubation at 37° C for 4 h, after which 1 mL of fresh medium was added to dilute Polybrene. After 24 h of culture, the medium containing viruses was replaced with fresh medium, and after 48 h of culture, the cell proteins were collected for Western blotting assay.

### Western blotting

The total proteins were extracted using a whole protein extraction kit (KeyGEN BioTECH, Nanjing, China), and the concentration was detected using a protein concentration detection kit. Then, the proteins were added with loading buffer and denatured in boiling water for 10 min. After sodium dodecyl sulphate-polyacrylamide gel electrophoresis (SDS-PAGE), the protein samples were transferred onto a membrane at a constant current of 300 mA, blocked with 0.5% skim milk at room temperature for 2 h, and incubated with primary antibodies against phosphorylated (p)-PI3K and total (t)-PI3K (CST, 17366S, diluted at 1: 800), p-mTOR and t-mTOR (ABCAM, ab109268, diluted at 1: 800), HIF-1α (Abcam, ab179483, diluted at 1: 1000), VEGFs (Proteintech,19003-1-AP, diluted at 1: 1000) at 4° C. On the next day, the protein samples were taken out and incubated with secondary antibodies at room temperature. Finally, densitometric analysis of the protein bands was performed.

### Quantitative polymerase chain reaction (qPCR)

The total RNAs were extracted from fresh tissues by TRIzol reagent. Briefly, 50 mg of tissues or cells frozen at -80° C were placed into an EP tube, ground quickly with grinding pestle, and then lysed with 1 mL of TRIzol reagent at room temperature for 5 min, followed by centrifugation at 12000 rpm for 1 min. Next, the supernatant was transferred to another new EP tube and gently mixed with 200 μL of chloroform, and the mixture was let stand at room temperature for 3 min. After centrifugation at 12000 rpm and 4° C for 10 min, white RNA precipitates appeared and were pasted into the EP tube. After the supernatant was discarded, the above procedure was repeated, if necessary, and the RNA precipitates were washed with 75% ethanol, and after vacuum drying, they were dissolved in DEPC-treated water. The concentration was measured by an UV spectrophotometer. Subsequently, the RNAs were immediately reversely transcribed into complementary deoxyribonucleic acids (cDNAs) under the 20 μL reverse transcription (RT) system: 10 μL of PrimeScript RT Enzyme Mix I, 1 μL of RT Primer Mix, 4 μL of 5 × PrimeScript buffer 2 (for Real Time) and 4 μL of DEPC-treated water. The PCR cycling parameters were pre-denaturation at 98° C for 2 min, followed by 40 cycles of denaturation at 95° C for 10 s, annealing at 55° C for 15 s and extension at 72° C for 30 s. The obtained cDNAs were subjected to qPCR under the reaction system (10 μL) as follows: 2 μL of cDNAs, 0.5 μL of upstream primers, 0.8 μL of downstream primers, 10 μL of 2× TransStart SYBR Green qPCR Supermix, 0.4 μL of ROX Reference Dye, and 6 μL of sterilized water. Each experimental group was set with 4 replicates, and each sample was set with 3 replicates. PCR reagents were purchased from TaKaRa.

### Endothelial tube formation assay

3B-11 and SVEC4-10 cells (2 endothelial cell lines) were cultured in an endothelial cell medium (ECM) containing 10% FBS and 1% double antibody, and the medium was changed every other day. When the cell fusion reached more than 80%, the cells were digested and subcultured with trypsin. Before the tube formation assay, Matrigel was melt at 4° C and paved into the precooled 24-well plate with a precooled pipette head, with 200μL/each well. After gentle shaking, the matrix glue was evenly distributed, and Matrigel was solidified at 37° C. Later, the two cell lines (5×10^5^ cells/mL) in the logarithmic growth phase were seeded into 24-well plates with 500 μL/well and cultured in the medium containing miR-29 inhibition lentivirus or PI-103, while those in control group were cultured in control medium. After 8 h, the cells were observed and pictured under the inverted microscope. Finally, the total numbers of branch nodes, rings and tubes of each image were quantified.

### Statistical analysis

Experiment data were obtained from at least 3 independent experiments and expressed as mean ± standard deviation (SD). Comparison of data between two groups was conducted using one-way analysis of variance (ANOVA) or unpaired *t* test. P<0.05 reported in two-way ANOVA represented that the difference was statistically significant. All statistical analyses were performed using GraphPad Prism software.
